# Is Emergency Department Care for Low Back Pain Meeting Contemporary Standards? A Medical Record Review

**DOI:** 10.1111/1742-6723.70214

**Published:** 2026-01-22

**Authors:** Pippa Flanagan, Piers Truter, Gustavo C. Machado, Kym Moiler, Rosalind Taylor, Paul Atkinson, Julie Bayliss, Ivan Lin, Karen Richards, Vinicius Cavalheri, Robert Waller

**Affiliations:** ^1^ Curtin School of Allied Health, Faculty of Health Sciences Curtin University Bentley Western Australia Australia; ^2^ Department of Physiotherapy Fiona Stanley Hospital Murdoch Western Australia Australia; ^3^ Emergency Department Fiona Stanley Hospital Murdoch Western Australia Australia; ^4^ School of Health Sciences and Physiotherapy The University of Notre Dame Fremantle Western Australia Australia; ^5^ Institute for Musculoskeletal Health The University of Sydney and Sydney Local Health District Sydney UK; ^6^ Western Australian Centre for Rural Health The University of Western Australia Geraldton Western Australia Australia; ^7^ Allied Health South Metropolitan Health Service Perth Western Australia Australia

**Keywords:** care standards, clinical guidelines, emergency department, low back pain, medical record review

## Abstract

**Background:**

In 2022, the Australian Commission on Safety and Quality in Healthcare released clinical care standards for managing low back pain (LBP), including in emergency departments (EDs), where guideline non‐adherent care has been reported. This study aimed to describe LBP care in a tertiary hospital ED against these new clinical standards.

**Methods:**

A 12‐month retrospective review of medical records from a random sample of adults with LBP presenting to a tertiary hospital ED. Outcomes focused on key aspects of LBP care aligned with the eight quality statements in the Low Back Pain Clinical Care Standards (LBPCCS). Descriptive statistics were used to describe care against the quality indicators.

**Results:**

Of the 1974 ED presentations with a LBP‐related discharge diagnosis in 2023, 550 were randomly selected for review. After exclusions, 374 were assessed against the LBPCCS. All presentations were assessed against the clinical assessment outcomes (quality statements 1–3), and 335 presentations were assessed against the ED management outcomes (quality statements 4–8). Clinical assessment and screening for serious/specific pathology was documented in 278 cases (74%); appropriate imaging in 66 (56%); self‐management advice in 56 (17%); and psychosocial risk screening with referral in 56 cases (17%). Opioids were administered in 208 cases (62%) and anti‐convulsants in 52 (16%).

**Conclusion:**

In a tertiary hospital ED, adherence to the LBPCCS is varied. The ED performed well against the clinical assessment/screening outcomes. However, findings suggest strategies are needed to improve performance against the management outcomes, including management advice, identification of psychosocial risk factors, and opioid use.

## 1 Introduction

Low back pain is a common reason people visit emergency departments (ED), accounting for 4.4% of ED visits internationally [1], and consistently being in the top‐five most common principal diagnoses for ED visits in Australia each year [2]. While people presenting to ED with LBP have a higher incidence of underlying serious spinal conditions than those presenting to primary care [3], 85% will have non‐serious LBP of non‐specific musculoskeletal cause [4]. Yet, ED LBP visits are often associated with extended length of stay (LOS) of > 4 h [4, 5] and costly hospital admissions [4], representing a considerable impact on the resource‐constrained healthcare system.

The care provided to people with LBP in ED is often not adherent to LBP guideline recommendations [6, 7, 8]. For example, it has been reported that 1 in 11 patients with LBP in ED receive an imaging test they did not need [9], and two‐thirds are prescribed at least one opioid medication [4], despite LBP guidelines consistently recommending against their routine use [10, 11]. Clinical practices that deviate from guideline‐based LBP care are frequent in ED, even though they have limited evidence of improving patient outcomes and have the potential to cause harm, such as unnecessary radiation [12], increased healthcare utilisation [13] and ongoing opioid use after discharge from ED [14]. Low‐value LBP care, such as inappropriate imaging and frequent use of opioid medications, can also negatively affect ED health service metrics, with studies reporting increased ED LOS and re‐presentation rates associated with their use [5, 15].

In 2022, Low Back Pain Clinical Care Standards (LBPCCS) were released to support healthcare services and clinicians in improving care for people with LBP [16]. The LBPCCS apply to adult patients presenting with an acute exacerbation of LBP and/or back‐related leg symptoms in all healthcare settings, including ED. Clinical care standards differ from clinical practice guidelines in that they recommend care within key areas for quality improvement rather than encompassing the entirety of LBP care. The LBPCCS focus on providing best practice care within the domains of clinical assessment (statements 1–3) and management, including review and referral (statements 4–8) (Table 1). The quality statements include suggested indicators for monitoring implementation and adherence within the healthcare setting, including specific recommendations for ED. Assessment of ED care for people with LBP against these suggested quality indicators has yet to be reported. Therefore, the aims of this study are to:

1. Describe the characteristics of adults presenting to a tertiary hospital ED with LBP according to their diagnostic categories.
2. Evaluate and describe the care provided to adults presenting to a tertiary hospital ED with LBP according to the LBPCCS quality indicators.

**TABLE 1:** LBPCCS quality statements and indicators for local monitoring [16].

Quality statement		Structure indicator	Process indicator
*Clinical assessment outcomes*			
1	Initial clinical assessment	1a. Evidence of a locally approved LBP assessment protocol	1b. Proportion of patients with acute LBP with the findings from both their initial clinical assessment and screening for specific and/or serious underlying pathology documented in their medical record
2	Psychosocial assessment	2a. Evidence of a locally approved policy to ensure that patients are screened for psychosocial factors early in each new presentation	
3	Reserve imaging for suspected serious pathology	3a. Evidence of a locally approved policy to ensure the appropriate use of imaging for LBP	3b. Proportion of patients with a new episode of LBP referred for imaging for whom an appropriate indication for imaging is documented in the medical record
*Management outcomes*			
4	Patient education and advice	4a. Evidence of local arrangements to ensure that patients are provided with information, advice and reassurance	
5	Encourage self‐management and physical activity		5a. Proportion of patients with LBP who have documented discussions in their medical record about both self‐management strategies and staying active by continuing usual activities
6	Physical and/or psychological interventions	6a. Evidence of a locally approved policy that specifies the referral pathways to clinicians who provide appropriate physical and/or psychological therapies	6b. Proportion of patients with LBP at risk of poor outcomes who were referred to physical and/or psychological clinical services
7	Judicious use of pain medicines		7a. Proportion of patients with LBP who received an opioid analgesic 7b. Proportion of patients with LBP who received an anticonvulsant
8	Review and referral	8a. Evidence of a locally approved policy that defines the process for review and referral of patients with LBP	

Abbreviation: LBP, low back pain.

## 2 Methods

### 2.1 Study Design

This was a retrospective observational analysis from a public tertiary hospital ED (110,000 presentations per annum) in Perth, Western Australia. A detailed description of the methods for this study (including patient and public involvement) can be found in the online Data S1.

### 2.2 Study Population

Emergency visit data from adult (≥ 18 years) presentations to ED with LBP between January 1 and December 31, 2023, were reviewed. International Classification of Diseases (ICD‐10) codes [17] (discharge diagnosis) were used to identify eligible LBP presentations to ED (Data S2: Table S1). During the review process, presentations that did not involve a lumbar spine condition were excluded (e.g., genitourinary presentations). Those who did not wait (DNW) were also excluded.

### 2.3 Sample Size

We used a formal approach for retrospective medical record review studies to determine the number of LBP presentations required to estimate our quality indicators with adequate precision [18]. Expected proportions for each indicator were informed by the LBPCCS quality indicators [16] and prior ED LBP studies [4, 8, 9], ranging between 15% and 60% of presentations. Using a standard precision‐based method for single proportions (95% confidence interval; desired half‐width ±0.05), these expected proportions correspond to required sample sizes between 196 and 369 ED LBP presentations. We therefore aimed to include at least 369 LBP presentations to ensure that all indicators within this expected range could be estimated with approximately ±0.05 precision (i.e., the true population proportion would differ from our estimate by no more than about five percentage points).

### 2.4 Data Collection

In anticipation of exclusion due to ineligibility upon review of medical records, a list of 550 presentations was generated using an online randomising tool [19]. A team of four ED clinician‐researchers (PF, PT, PA, JB) individually reviewed each medical record to extract LBP characteristics (e.g., pain duration, pain intensity, and patient function) and evidence of ED care against the LBPCCS quality statements (Table 1) [16]. If there was no documented information about a quality statement, it was assumed that the care was not provided.

### 2.5 Bias

To ensure consistency in data extraction, each case was initially reviewed independently by two researchers (PF and either PT, PA, or JB). Inconsistencies were discussed in consecutive group consensus meetings with a reference sheet developed to guide interpretation.

### 2.6 Variables

Outcomes were categorised as: (i) Patient demographics and ED visit outcomes; (ii) LBP characteristics (including function defined as ambulation capacity using the Functional Activity Scale (FAS) [20] (Data S2: Table S2)); and (iii) ED care against the LBPCCS process indicators aligned to the eight quality statements (Table 1) [16]. These indicators formed the primary outcomes, divided into clinical assessment (quality statements 1–3) and management outcomes (quality statements 4–8). Additional outcomes evaluated lumbar imaging use, including overuse (imaging without clinical indication) and underuse (no imaging despite clinical indication) [9, 21]. Imaging appropriateness was determined by the presence of alerting features for serious spinal pathologies as defined in the LBPCCS [16] (Data S2: Table S3).

### 2.7 Statistical Methods

The lumbar spine presentations were categorised into four groups (Data S2: Table S2): (i) Non‐specific LBP (NSLBP); (ii) LBP with radiating leg pain and/or neurological symptoms but no correlating neurological signs on physical examination (LBP with leg symptoms); (iii) LBP with radiating leg pain and/or neurological symptoms with correlating neurological signs on physical examination (LBP with radiculopathy); and (iv) LBP due to diagnoses of serious spinal pathology (e.g., spinal metastases). Serious spinal conditions were excluded from the management outcomes as they were considered irrelevant to certain aspects of LBP care (e.g., education on self‐management).

Except for imaging, outcomes were reported as the proportion of the total lumbar spine sample population. Imaging outcomes were reported as the proportion of the total imaging presentations as suggested by the imaging statement process indicator (Table 1). Descriptive analyses were used to report all outcomes independently. Categorical variables were reported as percentages, and continuous variables with normal distribution were reported as means (SD). Median and inter‐quartile range (IQR) were reported if data were not normally distributed.

## 3 Results

### 3.1 Sample Characteristics

There were 1974 ED presentations with an eligible ICD‐10 LBP discharge code identified. From the random sample of 550 presentations included for review, 176 presentations were excluded due to ineligibility. This left 374/550 (68%) presentations included in the assessment against the key outcomes (Data S2: Figure S1).

### 3.2 Demographic, ED Visit and LBP Characteristics

The demographic, ED visit and LBP characteristics of the presentations are described in Table 2. Overall, the median age of the lumbar spine sample was 51 years (IQR 37–67), and just over half of the presentations were from female patients (*n* = 194/374, 52%). One‐quarter of the presentations arrived by ambulance (*n* = 95/374, 25%), the median ED LOS was 5 h (IQR 3–8), and 258/374 (69%) were discharged home from the ED. Functional capacity was documented in 229/374 (61%) of presentations. Of these, 82/229 (36%) were classified as having no limitation in ambulation status, 124/229 (54%) having mild limitation to ambulation, and 23/229 (10%) with severe limitation to their ambulation status.

**TABLE 2:** Demographic, ED visit and LBP characteristics by lumbar spine diagnostic category.

	By lumbar spine diagnostic category				All lumbar spine presentations (*n* = 374)
	NSLBP (*n* = 194)	LBP with leg symptoms (*n* = 107)	LBP with Radiculopathy (*n* = 34)	Serious spinal pathology (*n* = 39)	
*Patient demographics*					
Age (years), median (IQR)	47 (33–64)	54 (38–69)	55 (46–64)	62 (43–78)	51 (37–67)
Gender, female, *n* (%)	85 (44)	64 (60)	19 (56)	26 (67)	194 (52)
*ED visit characteristics*					
Referred by, *n* (%)					
Self/relative	148 (76)	84 (79)	25 (74)	22 (56)	279 (75)
GP	46 (24)	23 (21)	9 (26)	17 (44)	95 (25)
Ambulance arrival, *n* (%)	46 (24)	22 (21)	9 (26)	18 (46)	95 (25)
Triage category, *n* (%)					
1	0 (0)	0 (0)	0 (0)	0 (0)	0 (0)
2	5 (3)	5 (5)	4 (12)	8 (21)	22 (6)
3	69 (36)	46 (43)	15 (44)	10 (26)	140 (37)
4	114 (59)	53 (50)	15 (44)	20 (51)	202 (54)
5	6 (3)	3 (3)	0 (0)	1 (3)	10 (3)
ED LOS (h), median (IQR)	5 (3–7)	5 (3–8)	5 (3–8)	8 (5–11)	5 (3–8)
ED pathway, *n* (%)					
Discharged	149 (77)	74 (69)	24 (71)	11 (28)	258 (69)
Emergency short stay	29 (15)	22 (21)	6 (18)	6 (15)	63 (17)
Inpatient ward	16 (8)	11 (10)	4 (12)	22 (56)	53 (14)
Treated in AECC	32 (16)	15 (14)	3 (9)	1 (3)	51 (14)
Treating clinician, *n* (%)*					
PCP	41 (21)	18 (17)	11 (32)	1 (3)	71 (19)
Junior Doctor	96 (50)	66 (62)	17 (50)	27 (69)	206 (55)
Senior Doctor	56 (29)	22 (21)	6 (18)	11 (28)	95 (26)
First ED LBP visit, *n* (%)	155 (80)	78 (73)	27 (79)	31 (80)	291 (78)
Visits in study year, *n* (%)					
Single LBP visit	165 (85)	76 (71)	27 (79)	29 (74)	297 (79)
2 or more ED visits	29 (15)	31 (29)	7 (21)	10 (26)	77 (21)
*LBP characteristics*					
Pain duration, *n* (%)					
Acute (0–6 weeks)	128 (66)	56 (52)	15 (44)	29 (74)	228 (61)
Subacute (7–12 weeks)	1 (1)	2 (2)	1 (3)	1 (3)	5 (1)
Chronic (> 12 weeks)	5 (3)	9 (8)	5 (15)	1 (3)	20 (5)
Acute or sub‐acute on chronic	60 (31)	40 (37)	13 (38)	8 (21)	121 (32)
Pain intensity*					
NPRS (0–10), med (IQR)	8 (7–10)	8 (7–10)	9 (6.5–10)	8 (7–10)	8 (7–10)
Function, *n* (%)					
Function not assessed	80 (41)	29 (27)	10 (29)	26 (67)	145 (39)
FAS 1 (no limitation)*	49 (43)	23 (29)	8 (33)	2 (15)	82 (36)
FAS 2 (mild limitation)*	55 (48)	48 (62)	12 (50)	9 (69)	124 (54)
FAS 3 (significant limitation)*	10 (9)	7 (9)	4 (17)	2 (15)	23 (10)

*Note:* *Missing data for treating clinician *n* = 2 (NSLBP *n* = 1; LBP with leg symptoms *n* = 1). *Missing data for pain intensity *n* = 35 (NSLBP *n* = 12; LBP with leg symptoms *n* = 4; radiculopathy *n* = 2; serious spinal pathology *n* = 17). *Denominator is total lumbar spine presentations with function documented *n* = 229 (NSLBP *n* = 114; LBP with leg symptoms *n* = 78; LBP with radiculopathy *n* = 24; Serious *n* = 13).

Abbreviations: AECC, Ambulatory Emergency Care Clinic; ED, emergency department; FAS, functional activity scale; GP, general practitioner; Junior Doctor, junior/resident medical officer or intern; NP, nurse practitioner; PCP, primary contact physiotherapist; Senior Doctor, registrar/senior registrar or consultant.

### 3.3 Compliance With Clinical Assessment Quality Indicators

All 374 presentations were included in the assessment of the clinical assessment outcomes. Overall, 278/374 (74%) of presentations had both the clinical assessment and screening for serious or specific underlying pathology documented in the medical record (indicator 1b, Table 3). Screening of all six serious pathologies listed in the LBPCCS occurred in 26/374 (7%) of presentations (Data S2: Figure S2). Screening for psychosocial factors was documented in 31/374 (8%) of all presentations (quality statement 2, Table 3). Overall, there were 118/374 (32%) presentations where at least one imaging test was received. Of those who received imaging (*n* = 118), 66 (56%) had an appropriate indication for immediate imaging documented (indicator 3b, Table 3). Of the presentations who did not receive imaging (*n* = 256), 47 (18%) had a documented alerting feature that could have warranted consideration of ED imaging. Six of these already had a confirmed diagnosis of serious pathology on arrival and did not require further imaging in ED. However, in two cases, minor alerting features were present and were initially diagnosed with NSLBP. Subsequent ED presentations documented in the medical record revealed underlying serious pathology (Data S2: Table S4).

**TABLE 3:** Assessment of ED LBP care against the clinical assessment outcomes by lumbar diagnostic category (quality statements 1–3).

	NSLBP (*n* = 194)	LBP with leg symptoms (*n* = 107)	LBP with radiculopathy (*n* = 34)	Serious spinal pathology (*n* = 39)	Total (*n* = 374)
**Quality Statement 1—Initial clinical assessment** **Indicator 1b: proportion of patients with acute low back pain with the findings of both their clinical assessment and screening for serious or specific underlying pathology documented**					
**Indicator 1b, *n* (%)**	**131 (68)**	**86 (80)**	**28 (82)**	**33 (85)**	**278 (74)**
Clinical assessment documented, *n* (%)	134 (69)	86 (80)	28 (82)	33 (85)	281 (75)
Screening for serious pathology, *n* (%)	182 (94)	102 (95)	34 (100)	39 (100)	357 (96)
Screening for specific pathology, *n* (%)	102 (52)	76 (72)	29 (83)	15 (37)	222 (59)
**Quality Statement 2—Psychosocial assessment** **Indicator 2a: evidence of a locally approved policy to ensure that patients are screened for psychosocial factors early in each new presentation**					
Screening for psychosocial factors, *n* (%)	13 (7)	12 (11)	3 (10)	3 (8)	31 (8)
**Quality Statement 3—Reserve imaging for suspected serious pathology** **Indicator 3b: proportion of patients with a new episode of low back pain referred for imaging for whom an appropriate indication for imaging is documented**					
Received any lumbar imaging, *n* (%)	58 (30)	18 (17)	11 (32)	31 (79)	118 (32)
X‐ray	50 (26)	13 (12)	6 (18)	22 (56)	91 (24)
CT	11 (6)	2 (2)	0 (0)	14 (36)	27 (7)
MRI	3 (2)	5 (5)	5 (15)	5 (13)	18 (5)
**Indicator 3b (appropriate imaging), *n* (%)** ^a^	**25 (43)**	**8 (44)**	**8 (73)**	**25 (81)**	**66 (56)**
Inappropriate imaging (overuse), *n* (%)^a^	33 (57)	10 (56)	3 (27)	6 (19)	52 (44)
Inappropriate no‐imaging (underuse), *n* (%)^b^	16 (12)	17 (19)	8 (35)	6 (75)	47 (18)

Abbreviations: CT, computed tomography; LBP, low back pain; MRI, magnetic resonance imaging; NSLBP, non‐specific low back pain.

^a^ Denominator is total who received imaging for each group *n* = 118 (total imaging) (NSLBP *n* = 58: LBP with leg symptoms *n* = 18; radiculopathy *n* = 11; serious pathology *n* = 31).

^b^ Denominator is total who received no‐imaging for each group *n* = 256 (total no‐imaging) (NSLBP *n* = 136; LBP with leg symptoms *n* = 89; radiculopathy *n* = 23; serious pathology *n* = 8).

### 3.4 Compliance With Management Outcome Quality Indicators

After excluding those with serious spinal conditions (*n* = 39), a total of 335 presentations were included in the assessment of the management outcomes (Table 4). Documented discussions about both self‐management strategies and staying active by continuing usual activities occurred in 56/335 (17%) of the presentations (indicator 5a). Psychosocial risk factors were evident in 117/335 (35%) of the included presentations, and 56/335 (17%) of these were referred to physical or psychological interventions (indicator 6a). At least one opioid medication was administered in 208/335 (62%) of presentations (indicator 7a). Anti‐convulsant medication was administered in 52/335 (16%) of presentations (indicator 7b).

**TABLE 4:** Assessment of ED LBP care against the management outcomes by lumbar diagnostic category (quality statements 4–8).

**Quality Statement 4—Patient education and advice (no process indicator for this statement)**				
**Quality Statement 5—Encourage self‐management and physical activity** **Indicator 5a: Proportion of patients with low back pain who had documented discussions in their medical record about both self‐management strategies and staying active by continuing usual activities**				
	NSLBP (*n* = 194)	LBP with leg symptoms (*n* = 107)	LBP with radiculopathy (*n* = 34)	Total (*n* = 335)
**Indicator 5a: *n* (%)**	**31 (16)**	**20 (19)**	**7 (21)**	**56 (17)**
**Quality Statement 6 – Physical and/or psychological interventions** **Indicator 6a: Proportion of patients with low back pain at risk of poor outcomes who were referred to physical and/or psychological clinical services**				
**Indicator 6a, *n* (%)**	**32 (16)**	**20 (19)**	**4 (12)**	**56 (17)**
Psychosocial risk factors present, *n* (%)	66 (34)	38 (36)	13 (38)	117 (35)
Referred, *n* (%)	82 (42)	61 (57)	12 (35)	155 (46)
**Quality Statement 7—Judicious use of pain medicines** **Indicator 7a: Proportion of patients with low back pain who received an opioid analgesic** **Indicator 7b: Proportion of patients with low back pain who received anticonvulsants**				
Received analgesia in ED, *n* (%)	167 (86)	96 (93)	27 (79)	290 (87)
**Indicator 7a (opioids), *n* (%)**	**111 (57)**	**77 (72)**	**20 (59)**	**208 (62)**
**Indicator 7b (anticonvulsants), *n* (%)**	**11 (6)**	**31 (29)**	**10 (29)**	**52 (16)**
**Quality Statement 8—Review and referral (no process indicator for this statement)**				

Abbreviations: LBP, low back pain; NSLBP, non‐specific low back pain.

## 4 Discussion

### 4.1 Statement of Principal Findings

One year after the release of the Australian LBPCCS, care for patients with LBP in a tertiary ED remains non‐adherent to many key quality indicators. Most patients received an appropriate initial assessment, and one‐third underwent imaging. However, only half of those imaged had a documented indication supporting the need for ED imaging. Among those not imaged, a small proportion had documented alerting features that might have warranted consideration of ED imaging. Despite being discouraged by the LBPCCS, opioid use remains common, whereas active strategies promoting self‐management are underused. In the ED setting, quality improvement is needed across most key areas of LBP care outlined in the LBPCCS.

### 4.2 Strengths and Limitations of This Study

The use of ICD‐10 discharge codes to identify lumbar spine populations in ED can miss eligible presentations when coding is incorrect. To mitigate this, the multidisciplinary research team developed a comprehensive list of all codes that could reasonably be used by ED clinicians for patients presenting with LBP (Table S1; Data S2). This captured over 1900 presentations in the study year and, while a small number of missed cases remain possible, we are confident that most eligible presentations were identified. A key strength of this study was the comprehensive review of all documentation related to the full ED episode of care, allowing an in‐depth analysis of care. However, due to the retrospective nature of the medical record review, interpretation of care was limited to what was documented. With aspects of ED care that are potentially not as well‐documented (e.g., discussions on self‐management), the low adherence to these quality indicators may reflect documentation issues rather than provision of low‐value care. The manual audit methods employed increased the time required to review some individual cases, resulting in only 550 of more than 1900 LBP presentations being included for review. However, a rigorous sample‐size calculation confirmed that the 374 included presentations were sufficient to ensure an appropriate and representative analysis. The time required for record review also limited formal inter‐observer reliability analysis; however, the use of a reference guide and group review of unclear cases helped ensure consistency and minimise bias.

Although conducted at a single Australian tertiary ED, patient characteristics align with prior Australian [4, 7, 8] and international ED LBP studies [22, 23], suggesting the findings are likely to be generalisable to other similar settings.

### 4.3 Comparison to Other Studies

A large retrospective analysis of routinely collected electronic medical record data from three metropolitan ED's in Sydney, Australia has been published [4]. This study used automated data extraction from the medical record, enabling efficient collection of healthcare data and a substantial sample size (*n* = 6393). Like our study, this research highlights the burden of LBP on the health system, with prolonged ED LOS, high admission rates and ambulance use. While automated data collection limited detailed analysis of ED care, similar rates of opioid use and lumbar imaging were observed. Recent studies assessing ED adherence to LBP guidelines in Australian ED's used manual review methods, like ours [6, 7, 8], but often applied broad or mixed guideline criteria, limiting comparability of findings. For example, lumbar imaging appropriateness was reported as high as 98% [8], compared to only 56% in our study, which is likely due to differing criteria used. A strength of our study was the use of rigorous criteria based strictly on the LBPCCS process indicators, facilitating comparability and reproducibility. Another study from Sydney, Australia using strict criteria also reported 56% of imaging referrals were either appropriate or potentially appropriate aligning with our results [9]. Despite methodological differences, studies consistently highlight key gaps in ED LBP care, including incomplete screening for serious pathologies [7, 8], inappropriate imaging use [9], limited consideration of psychosocial risk factors [7], and a preference for pharmacological over non‐pharmacological treatments [6, 7].

### 4.4 Possible Explanations and Implications for Clinicians and Policymakers

At face value, there appears to be acceptable adherence to the clinical assessment outcomes. This is consistent with other ED LBP research [7, 8] and likely reflects the frequency with which patients with LBP in ED are screened for well‐known serious pathologies such as cauda equina syndrome. However, only 7% of patients had documented full screening of all serious pathologies listed in the LBPCCS, suggesting critical screenings were frequently missed or not documented. This may partly account for the observed rates of imaging overuse (undocumented alerting feature) and underuse (missed indications), as demonstrated by two presentations that later re‐presented with serious pathology. These cases also highlight the limited diagnostic value of isolated alerting features in the ED setting [3] and underscore the importance of complete screening and consideration of combinations of alerting features. Psychosocial risk screening is infrequently performed in ED, likely due to the lack of efficient, validated tools suitable for use in this setting [24]. Emergency settings may benefit from implementing simplified screening methods or developing ED‐specific tools to identify patients at risk of poor outcomes.

The low adherence to the management outcomes may be attributed to patient‐related factors unique to ED. Using the Functional Activity Scale, we identified distinct subsets of ED patients with LBP based on their ambulation capacity. Ambulation was chosen to define functional capacity as this is an essential consideration for safe discharge from the ED. While many patients present to ED without significant ambulation limitations, a small subset experiences severe pain preventing movement from the hospital bed. This latter group is often the most challenging to provide guideline‐recommended care for, as their severe symptoms typically necessitate pharmacological intervention. The higher severity of symptoms in patients with LBP in ED [25] and clinicians feeling they “need to do something” have been reported as barriers to improving opioid use in patients with LBP in the ED setting [26].

In the complex ED environment, deviations from the standards of care outlined in the LBPCCS can have implications for the patient and the health service. Patients risk having serious pathology missed from incomplete screening and potential harm associated with unnecessary imaging tests and opioid use [5, 12, 13]. For health services, consequences include resource wastage and negative impact on metrics such as increased LOS [5]. Future research should evaluate strategies to implement key LBPCCS care components in the ED setting.

### 4.5 Future Research and Unanswered Questions

Optimal strategies to implement the Australian LBPCCS in the ED setting are uncertain [27]. Based on our findings, future strategies to improve clinical assessment for patients with LBP should focus on complete screening for all serious pathologies, more appropriate use of imaging (particularly X‐ray), and better identification of psychosocial risk factors that may affect recovery. Changes to improve documentation in the medical record, including embedded checklists for serious pathology screening, psychosocial screening, and indications for lumbar imaging, should be considered. Future strategies to improve LBP management outcomes in ED should focus on enhancing patient education and advice on self‐management and optimising the use of opioid medications. Objective measures of functional capacity should be incorporated in the ED to identify LBP subgroups based on symptom severity and ambulation capacity. This would allow for care pathways to be tailored to the patient's needs based on their presenting symptoms. For example, in patients with the most severe symptoms, efforts to optimise care could focus on improving opioid prescribing practices, such as risk screening and tapering advice, rather than simply reducing use. However, clearer guidance on appropriate opioid use for LBP is still needed. To improve comparability of findings across studies within the same country, future research should adopt consistent guidelines with clear and reproducible criteria to assess the care provided to people with LBP in ED.

## 5 Conclusion

One year after the release of the new Australian Low Back Pain Clinical Care Standards, variable adherence to evidence‐based care recommendations for people with LBP persists in the emergency setting. Patients with LBP in ED are more likely to receive imaging and medication than the recommended non‐pharmacological care, such as reassurance and self‐management advice. Strategies are needed to address the challenges of implementing the new LBP standards in emergency settings.

## Author Contributions

The corresponding author (PF) confirms that all individuals listed as authors meet the established authorship criteria and that no others meeting this criterion have been excluded.

## Funding

This study received a WA Health Translation Network Enabling Allied Health Research Capacity 2024 grant provided by the Chief Allied Health Office through the Allied Health Enabling Platform.

## Ethics Statement

Ethics approval was obtained from the South Metropolitan Health Service Human Research Ethics Committee (protocol number RGS0000005819) and the Curtin University Human Research Ethics Office (approval number HRE2023‐0367).

## Conflicts of Interest

The authors declare no conflicts of interest.

## Supporting information


**Data S1:** Supporting Information.

**Data S2:** Supporting Information.

## Data Availability

The data that support the findings of this study are available from the corresponding author upon reasonable request.
