# Supplementary material for: Is Emergency Department Care for Low Back Pain Meeting Contemporary Standards? A Medical Record Review
Source: Emerg Med Australas. 2026 Jan 22;38(1):e70214. doi: 10.1111/1742-6723.70214 (PMC12828247; doi:10.1111/1742-6723.70214)
Supplement: Supplementary file 1 — Data S1: Supporting Information. [file EMM-38-0-s001.docx]

**METHODS**

**Study design**

This was a retrospective observational analysis using routinely collected health data from a public tertiary hospital in Australia. The study was conducted and reported according to the STROBE (Strength of Reporting in Observational Studies in Epidemiology) Statement and its extension statement RECORD (REporting of studies Conducted using Observational Routinely collected health Data) [1]. Ethics approval, including a waiver of consent, was obtained from the South Metropolitan Health Service Human Research Ethics Committee (protocol number RGS0000005819) and the Curtin University Human Research Ethics Office (approval number HRE2023-0367).

**Patient and Public Involvement**

Members of the public were involved in the design of this study through consultation with the Emergency Department (ED) Consumer Advisory Group. The group provided feedback on the audit data collection tool, with particular focus on the language used as evidence of psychosocial risk factors. Their feedback informed revisions to ensure the tool captured meaningful and patient-centred outcomes.

**Setting**

The setting is the second busiest ED in Australia, with over 110,000 patient presentations each year [2]. The ED is within a teaching tertiary hospital with junior medical officers rotating every three months and registrars every six months as part of their training. The ED multidisciplinary team includes nurses, nurse practitioners, and allied health (including primary contact physiotherapists). The ED includes an Emergency Short-stay Unit (ESSU) and, during the study period, also had an established Ambulatory Emergency Care Centre (AECC) staffed by the Acute Medical Unit (AMU), where suitable patients are diverted from the ED to a co-located outpatient clinic (Figure 1).


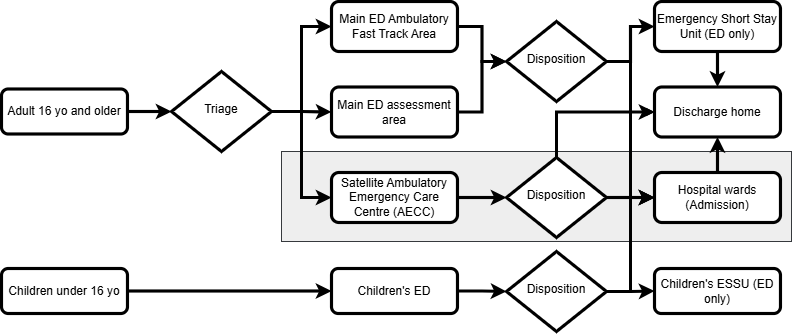


**Figure 1**: Study setting, including satellite Ambulatory Emergency Care Centre (AECC) pathway

*ESSU = emergency short stay unit

**Study population**

Emergency visit data from adult (≥18 years) presentations to ED with LBP between January 1 and December 31, 2023, were reviewed. International Classification of Diseases (ICD-10) codes [3] (discharge diagnosis) were used to identify eligible LBP presentations to ED (online supplementary file 2: Table S1). This included presentations related to serious spinal conditions (e.g. vertebral fracture) and LBP-related radicular syndromes (e.g. radiculopathy). Multiple presentations for the same patient were included as discrete episodes of ED care eligible for individual medical record review. The ED episode of care was defined as the time the patient spent in the ED from triage to the time they were discharged from the ED or admitted to the hospital. Care provided within the ESSU and AECC was considered part of the ED episode of care, with data from these admissions included in the medical record review. Data from inpatient ward admissions were not included. During the review process, presentations that did not involve a lumbar spine condition were excluded (e.g. genitourinary presentations). Those who did not wait (DNW) were also excluded.

**Data sources**

Data were extracted from Web Patient Administration System (webPAS), the Emergency Department Information System (EDIS), and the patient's digital medical record. Other data sources included InteleConnect (imaging database) and I.Clinical Manager (investigations and medication prescription). Data were collected from triage notes, nursing clinical notes, medical imaging request forms, medication charts, and digital medical records.

**Sample size**

We used a formal approach for retrospective medical record review studies to determine the number of LBP presentations required to estimate our quality indicators with adequate precision [4]. Expected proportions for each indicator were informed by the LBPCCS quality indicators [5] and prior ED LBP studies [6–8], ranging between 15% to 60% of presentations. Using a standard precision-based method for single proportions (95% confidence interval; desired half-width ±0.05), these expected proportions correspond to required sample sizes between 196 and 369 ED LBP presentations. We therefore aimed to include at least 369 LBP presentations to ensure that all indicators within this expected range could be estimated with approximately ±0.05 precision (i.e., the true population proportion would differ from our estimate by no more than about five percentage points).

**Data collection**

Data from eligible presentations were de-identified, and demographic and ED visit data were extracted onto a securely stored Excel spreadsheet. In anticipation of exclusion due to ineligibility upon review of medical records, a list of 550 presentations was generated using an online randomising tool [9]. The data for each presentation were uploaded as individual case records into a Research Electronic Data Capture (REDCap) database developed for the study [10]. The database was developed by two study authors (PF and PT) with consultation from the multidisciplinary ED clinical team (KM) and ED consumers via the study ED consumer advisory group.

Patient demographic and ED visit data (e.g. ED LOS) were automatically extracted from Webpas and EDIS into REDCap. A team of four ED clinician-researchers (PF, PT, PA, JB) then individually reviewed each medical record to extract LBP characteristics (e.g. pain duration, pain intensity, and patient function) and evidence of ED care against the LBPCCS quality statements (Table 1) [5]. If there was no documented information about a quality statement, it was assumed that the care was not provided.

**Table 1**: LBPCCS quality statements and indicators for local monitoring [5]

| **Quality Statement** | | **Structure Indicator** | **Process Indicator** |
| --- | --- | --- | --- |
| **Clinical assessment outcomes** | | | |
| 1 | Initial clinical assessment | 1a. Evidence of a locally approved LBP assessment protocol | 1b. Proportion of patients with acute LBP with the findings from both their initial clinical assessment and screening for specific and/or serious underlying pathology documented in their medical record |
| 2 | Psychosocial assessment | 2a. Evidence of a locally approved policy to ensure that patients are screened for psychosocial factors early in each new presentation |  |
| 3 | Reserve imaging for suspected serious pathology | 3a. Evidence of a locally approved policy to ensure the appropriate use of imaging for LBP | 3b. Proportion of patients with a new episode of LBP referred for imaging for whom an appropriate indication for imaging is documented in the medical record |
| **Management outcomes** | | | |
| 4 | Patient education and advice | 4a. Evidence of local arrangements to ensure that patients are provided with information, advice and reassurance |  |
| 5 | Encourage self-management and physical activity |  | 5a. Proportion of patients with LBP who have documented discussions in their medical record about both self-management strategies and staying active by continuing usual activities |
| 6 | Physical and/or psychological interventions | 6a. Evidence of a locally approved policy that specifies the referral pathways to clinicians who provide appropriate physical and/or psychological therapies | 6b. Proportion of patients with LBP at risk of poor outcomes who were referred to physical and/or psychological clinical services |
| 7 | Judicious use of pain medicines |  | 7a. Proportion of patients with LBP who received an opioid analgesic  7b. Proportion of patients with LBP who received an anticonvulsant |
| 8 | Review and referral | 8a. Evidence of a locally approved policy that defines the process for review and referral of patients with LBP |  |

Abbreviation: LBP – Low back pain.

**Bias**

To ensure consistency in data extraction, each case was initially reviewed independently by two researchers (PF and either PT, PA, or JB). Inconsistencies were discussed in consecutive group consensus meetings with a reference sheet developed to guide interpretation. If there was ongoing uncertainty during the review process, a field comment was left to flag a second opinion (PF), with escalation to group consensus as required. After initial review, if a presentation was deemed ineligible due to not involving a lumbar spine condition, the case record was flagged and reviewed by a second researcher before being excluded. The reasons for excluding cases were recorded.

**Variables**

Outcomes were divided into: (i) Patient demographics and ED visit outcomes, (ii) LBP characteristics (including function defined as ambulation capacity using the Functional Activity Scale (FAS) [11]) (online supplementary file 2: Table S2), and (iii) ED care against the LBPCCS process indicators aligned to the eight quality statements (Table 1) [5]. The process indicators informed the key study outcomes, which were divided into clinical assessment (quality statements 1-3) and management outcomes (quality statements 4-8). The structure indicators were not included as outcomes as the study ED did not have LBP policies during the study period. Further outcomes related to lumbar imaging were included to explore the context in which imaging practices were used in ED. This included imaging overuse (imaging without a clinical indication for immediate/ED imaging) and imaging underuse (no imaging with an appropriate clinical indication for immediate/ED imaging) [6,12]. The criterion for determining imaging appropriateness was based on the presence of alerting features listed for each spinal pathology in the LBPCCS. As such, only serious pathologies where immediate imaging is recommended were included as criterion for imaging appropriateness [5] (online supplementary file 2: Table S3).

**Statistical methods**

The lumbar spine presentations were categorised into four groups (online supplementary file 2: Table S2): (i) Non-specific LBP (NSLBP); (ii) LBP with radiating leg pain and/or neurological symptoms but no correlating neurological signs on physical examination (LBP with leg symptoms); (iii) LBP with radiating leg pain and/or neurological symptoms with correlating neurological signs on physical examination (LBP with radiculopathy); and (iv) LBP due to diagnoses of serious spinal pathology (e.g. spinal metastases). Four diagnostic categories were reported as neurological findings on clinical examination may influence ED care (e.g. review and referral). Axial spondyloarthropathies were included in the serious spinal pathology group [5]. All four diagnostic categories were included in the clinical assessment outcomes. However, serious spinal conditions were excluded from the management outcomes as they were considered irrelevant to certain aspects of LBP care, including education on self-management, physical activity advice, and pharmacological treatment.

Except for imaging, outcomes were reported as the proportion of the total lumbar spine sample population. Imaging outcomes were reported as the proportion of the total imaging presentations as suggested by the imaging statement process indicator (Table 1). Descriptive analyses were used to report all outcomes independently. Categorical variables were reported as percentages, and continuous variables with normal distribution were reported as means (SD). Median and inter-quartile range (IQR) were reported if data was not normally distributed.

References

1 Benchimol EI, Smeeth L, Guttmann A, *et al.* The REporting of studies Conducted using Observational Routinely-collected health Data (RECORD) Statement. *PLOS Medicine*. 2015;12:e1001885. doi: 10.1371/journal.pmed.1001885

2 Keane C, Clayden V, Scott G. Evaluation of an Ambulatory Emergency Care Centre at a tertiary hospital in Perth, Western Australia. *Australasian Emergency Care*. 2022;25:289–95. doi: 10.1016/j.auec.2022.02.001

3 ICD-10 Version:2010. https://icd.who.int/browse10/2010/en (accessed 15 January 2025)

4 Johnston KM, Lakzadeh P, Donato BMK, *et al.* Methods of sample size calculation in descriptive retrospective burden of illness studies. *BMC Med Res Methodol*. 2019;19:9. doi: 10.1186/s12874-018-0657-9

5 Low Back Pain Clinical Care Standard | Australian Commission on Safety and Quality in Health Care. https://www.safetyandquality.gov.au/standards/clinical-care-standards/low-back-pain-clinical-care-standard (accessed 11 December 2024)

6 Traeger AC, Machado GC, Bath S, *et al.* Appropriateness of imaging decisions for low back pain presenting to the emergency department: a retrospective chart review study. *International Journal for Quality in Health Care*. 2021;33:mzab103. doi: 10.1093/intqhc/mzab103

7 Ferreira GE, Machado GC, Shaheed CA, *et al.* Management of low back pain in Australian emergency departments. *BMJ Qual Saf*. 2019;28:826–34. doi: 10.1136/bmjqs-2019-009383

8 Heine J, Window P, Hacker S, *et al.* Adherence to recommended guidelines for low back pain presentations to an Australian emergency department: Barriers and enablers. *Australasian Emergency Care*. 2023;26:326–32. doi: 10.1016/j.auec.2023.04.003

9 RANDOM.ORG - True Random Number Service. https://www.random.org/ (accessed 7 January 2025)

10 Harris PA, Taylor R, Minor BL, *et al.* The REDCap consortium: Building an international community of software platform partners. *Journal of Biomedical Informatics*. 2019;95:103208. doi: 10.1016/j.jbi.2019.103208

11 Levy N, Sturgess J, Mills P. “Pain as the fifth vital sign” and dependence on the “numerical pain scale” is being abandoned in the US: Why? *British Journal of Anaesthesia*. 2018;120:435–8. doi: 10.1016/j.bja.2017.11.098

12 Yates M, Oliveira CB, Galloway JB, *et al.* Defining and measuring imaging appropriateness in low back pain studies: a scoping review. *Eur Spine J*. 2020;29:519–29. doi: 10.1007/s00586-019-06269-7
