# Supplementary material for: Is Emergency Department Care for Low Back Pain Meeting Contemporary Standards? A Medical Record Review
Source: Emerg Med Australas. 2026 Jan 22;38(1):e70214. doi: 10.1111/1742-6723.70214 (PMC12828247; doi:10.1111/1742-6723.70214)
Supplement: Supplementary file 2 — Data S2: Supporting Information. [file EMM-38-0-s002.docx]

| Code | Diagnosis |
| --- | --- |
| C41.2 | Vertebral column cancer |
| C79.9 | Neoplasia – metastases multiple |
| C80.9 | Neoplasia – multiple metastases |
| G83.4 | Cauda equina syndrome |
| G95.2 | Neurological – peripheral – spinal cord compression |
| G95.2 | Cord compression, unspecified |
| M46.3 | Infection of intervertebral disc (pyogenic) |
| M51.0 | Lumbar and other intervertebral disc disorders with myelopathy |
| M51.2 | Other specified intervertebral disc displacement, back pain due to displacement of IV-disc, sciatica due to displaced IV disc |
| M53.3 | Sacroiliac pain |
| M53.3 | Sacrococcygeal disorders, no elsewhere classified |
| M54.19 | Orthopaedic - radiculopathy |
| M54.3 | Sciatica |
| M54.5 | Low back pain, low back strain, orthopaedic low back pain, lumbago |
| M54.99 | Back pain |
| M56.4 | Discitis, unspecified |
| S20.80 | Injury – bruise/contusion – back/spine central thoracolumbar, other superficial injuries |
| S32.0 | "Null" ?fracture diagnosis |
| S32.00 | Injury - fractures/closed – back/spine (left sided, lumbar vertebra, right sided, thoracolumbar) |
| S32.2 | Injury – fractures/closed – back/spine coccyx |
| S33.50 | Sprain and strain of lumbar spine, unspecified |
| S33.51 | Injury – sprain/strain – back/spine – coccyx pain |
| S33.7 | Injury – sprain/strain – back/spine – (coccyx, lower/lumbar, lumbosacral spine sprain/strain, sacrum, other and unspecified) |
| S34.3 | Injury of Cauda Equina |
| T08.0 | Injury - fractures/closed – back/spine other part |
| T09.2 | Injury – sprain/strain – back/spine – (back muscle spasm, other area) |

**Table S1**: included ICD-10 codes and diagnoses

| Lumbar spine diagnostic categories | 1. NSLBP = axial low back pain without radiation of pain or neurological signs/symptoms to the lower limbs 2. LBP with leg symptoms = NSLBP with radiation of pain or other sensory symptoms to the lower limbs but no physical examination findings of nerve involvement (dermatomal sensory changes, reflex changes, or myotomal power loss) 3. Radiculopathy = LBP with typically unilateral leg pain and at least one of the associated symptoms of: dermatomal paraesthesia/numbness, true motor weakness, or reduced lower limb reflexes (+/- correlating MRI findings) 4. Serious spinal pathology = LBP from serious spinal conditions listed in Table 1 of LBPCCS (Spinal infection, vertebral fracture, malignancy, visceral disease, suspected or threatened cauda equina compression, spinal cord pathology (myelopathy), axial spondyloarthritis) |
| --- | --- |
| Pain duration | Acute = 0-6 weeks  Sub-acute = 7-12 weeks  Chronic = >12 weeks  Acute or sub-acute on chronic = acute episode per above durations on the background of chronic or recurring LBP |
| Function | Ambulation status (can the patient ambulate?) |
| Functional Activity Scale (FAS) | The FAS was used to quantify functional capacity using a three-tier ambulation assessment:   1. No limitation (patient can ambulate without obvious signs of discomfort) 2. Mild limitation (patient can ambulate but shows moderate to severe pain e.g. pain-affected gait) 3. Significant limitation (patient cannot ambulate due to pain) |

**Table S2**: Definitions – Low Back Pain characteristics including diagnoses

NSLBP = non-specific low back pain; LBP = low back pain

| Clinical assessment outcome definitions | |
| --- | --- |
| Clinical assessment | Yes = full patient history and adequate physical examination documented  No = missing important aspects of history/examination, e.g. medical record did not include medication history |
| Radicular symptoms | Evidence in the medical record that patient had either pain or sensory symptoms radiating from their back to their legs |
| Serious pathologies and alerting features | Spinal infection – symptoms and signs of infection (fever), raised inflammatory markers (C-reactive protein, erythrocyte sedimentation rate), risk factors for infection (underlying disease, immunosuppression, penetrating wound, history of intravenous drug use)  Vertebral fracture – history of considerable trauma (see below), history of minor trauma (fall from standing height) if patient is older than 50 years or has a history of osteoporosis or taking corticosteroids (>5mg for >3 months)  Cancer (major risk) – history of myeloma or cancer  Cancer (minor risk) – unexplained weight loss/decreased appetite/fatigue, pain worse in supine position or rest (may be referred to as “unremitting night pain”), progressive multisite pain  Visceral disease – flank/abdominal pain, particularly with radiation to the back, associated collapse or hypotension, absence of aggravating features (non-mechanical pain)  Suspected or threatened cauda equina compression – changes to bladder or bowel function, reduced saddle/perianal sensation or saddle paraesthesia’s  Spinal cord pathology (myelopathy) – progressive bilateral lower limb weakness, clumsy/ataxic gait with reduced balance of potential falls, bilateral paraesthesia of lower limbs, clonus/positive Babinski, upper limb clumsiness/reduced fine motor control  Axial spondyloarthropathies – age younger than 40 years, symptom duration of more than 3 months, prolonged morning stiffness and night pain, alternating buttock pain, improvement of symptoms with physical activity or exercise, and failure to improve with rest and NSAIDs |
| Serious pathology screened | Yes = documented screening of at least one serious pathology listed above as evidenced by reported presence or absence of alerting features. Reported negative findings are considered as evidence of screening (e.g. “no history of trauma” is considered as screening for vertebral fracture)  Special considerations:   - Spinal infection was screened if observations were documented and the treating ED clinician acknowledged these in the physical examination findings (e.g. “afebrile”) - Visceral disease was screened if a urinalysis was done and acknowledged by the treating clinician - Cancer was screened if this was listed as part of their medical history - Myelopathy was screened if the subjective examination documented screening for the appropriate myelopathy alerting features AND physical examination included assessment of gait, power, LMN, and UMN reflexes and sensation. - Gait for neurological screening must include details on type of gait observed (i.e. “ambulated into department” was not considered as neurological gait screening)   No = no documented evidence that screening was undertaken (i.e. no alerting features are listed and negative findings such as “no history of cancer” are not reported) |
| Considerable trauma | Trauma A criteria = MVA > 60kph, MBA > 30kph (includes eScooter), ejection from vehicle, fall >3m, pedestrian/cyclist v car, fatality in vehicle, excessive cabin intrusion |
| Specific pathology screened | Specific pathologies = spinal stenosis, radiculopathy, axial spondyloarthropathies or “other” such as spondylolisthesis  Spinal stenosis screening = reported LBP with leg pain (typically bilateral) that is worse with prolonged standing/walking and eases with sitting/bending forward +/- sensory changes in limbs  Radiculopathy screening = the subjective examination documented screening for radiation of symptoms to the lower limbs AND physical examination included assessment of power, reflexes, and sensation  Axial spondyloarthropathy screening = consideration of this in documented medical history as well as above alerting features (note axial spondyloarthropathies were considered serious pathology per the LBPCCS)  Yes = evidence of any of the above findings in medical record (including imaging reports if the treating clinician acknowledges these)  No = no documented evidence that screening was undertaken (i.e. no alerting features are listed and negative findings such as “no history of cancer” are not reported) |
| Psychosocial factors screened | Yes = documented evidence of positive or negative psychosocial findings per listed risk factors associated with delayed recovery listed in the LBPCCS:   - Belief that pain and activity are hamful - “sickness” behaviours (such as extended rest) - Low or negative moods, or social withdrawal - Mental health comorbidities or trauma history - Treatment that does not fit with best practice, but remains the focus of the patient’s requests - Problems with the compensation system - Previous history of back pain, time off work or other claims - Problems at work or poor job satisfaction - Financial hardship - Overprotective family or lack of social support   No = no documented evidence that screening was undertaken (i.e. no risk factors are listed and negative findings such as “no relevant psychosocial history” is not reported) |
| Appropriate indications for immediate/ED imaging | CES – new urinary retention/faecal incontinence (changes to bladder/bowel function), saddle anaesthesia (reduced saddle/perianal sensation or saddle paraesthesis)  Infection – symptoms and signs of infection (fever, night sweats or systemically unwell), risk factors for infection (immunosuppression, intra-venous drug use, open wound, indwelling vascular catheter, other infection site), raised inflammatory markers  Vertebral fracture – history of considerable trauma (see above), minor trauma if >50, history of osteoporosis or prolonged corticosteroid use (>5mg for > 3 months)  Severe neurological deficit – progressive bilateral lower limb weakness/motor deficits at multiple neurological levels  Cancer (major risk) – history of cancer or myeloma |
| Management outcome definition | |
| Documented evidence of self-management strategies and advice to stay active | Accepted self-management strategies:   - Staying active - Move with confidence (reassurance that movement is ok) - Positive mindset - Set activity goals (including pacing) - Staying involved - Sleep habits - Vary posture (sit and stand comfortably) - Heat packs - Pain medication advice has to include education that they are used to stay active but not to completely stop pain   Activity advice:   - Returning to or continuing usual activity as soon as possible - Work advice - Exercise and physical activity advice - Pacing strategies   Note simply saying provided LBP pamphlet is not sufficient evidence of self-management advice |
| Non-opioid analgesics | Paracetamol  NSAIDs  Inhaled analgesic (nitrous/methoxyflurane)  Gabapentanoids (anticonvulsants = gabapentin/pregabalin)  Tricyclic antidepressants (amitriptyline, nortriptyline)  Serotonin and noradrenaline reuptake inhibitors (SNRIs = duloxetine, venlafaxine)  Benzodiazapines  Lignocaine patch |
| Opioid analgesics | Codeine  Tramadol  Tapentadol  Oxycodone  Targin (Oxycodone.naloxone)  Buprenorphine  Fentanyl  Morphine |

**Table S3**: Definitions – clinical assessment and management toutcomes

*LBPCCS = CES – cauda equina syndrome; Low back pain clinical care standards; *LMN = lower motor neurone; MBA = motorbike accident; MVA = motor vehicle accident; NSAIDS = non-steroidal anti-inflammatories; UMN = upper motor neurone

**Figure S1:** Flow chart of included presentations

*LBP = low back pain, NSLBP = non-specific low back pain

**Figure S2**: Number of serious pathologies screened in each medical record

| Patient demographics | Indexed ED diagnosis | Alerting features during indexed ED visit | ED investigations during indexed ED visit | Outcome |
| --- | --- | --- | --- | --- |
| 89-year-old male | NSLBP | Subjective fevers  Flank pain | Physiological observations within normal limits  Urinalysis unremarkable | Re-presented to ED the following day febrile, tachycardic, abdominal pain. Diagnosis of urosepsis. Admitted to the hospital for treatment. |
| 90-year-old male | NSLBP | Recent spinal injection (no systemic signs or symptoms) | Physiological observations within normal limits  Bloods unremarkable | Re-presented to ED 2 days later with increasing back and leg pain. Admitted to the hospital for management of pain. Abnormal bloods as inpatient with MRI confirming lower lumbar intraspinal infection, predominantly phlegmon with small abscess. Remained in hospital for treatment |

**Table S4:** Details of adverse consequences
